# Artificial Intelligence in Particle Therapy Treatment Planning: Current Advances and Future Directions

**DOI:** 10.1016/j.ijpt.2026.101954

**Published:** 2026-08-13

**Authors:** James C.L. Chow, Huan Giap

**Affiliations:** 1 Department of Medical Physics, Princess Margaret Cancer Centre, University Health Network, Toronto, Canada; 2 Department of Radiation Oncology, University of Toronto, Toronto, Canada; 3 Department of Materials Science and Engineering, University of Toronto, Toronto, Canada; 4 Nevada Oncology Specialists, Las Vegas, NV

**Keywords:** Artificial intelligence, Machine learning, Deep learning, particle and heavy ion therapy, Radiation treatment planning, Monte Carlo simulation, Hybrid AI and physical model

## Abstract

**Purpose:**

This review examines the role of artificial intelligence (AI) in particle therapy treatment planning, highlighting recent advancements, clinical potential, and existing challenges.

**Methods and Materials:**

A review was conducted on AI applications in proton and heavy ion therapy, focusing on automated plan generation, dose prediction, treatment adaptation, and quality assurance.

**Results:**

AI-driven methods have shown promise in optimizing treatment planning, enhancing dose prediction, and improving plan adaptation. However, challenges such as data limitations, model interpretability, and regulatory barriers hinder clinical implementation.

**Conclusion:**

While AI offers significant potential to improve particle therapy treatment planning, further research is needed to enhance model robustness, clinical validation, and integration into existing workflows.

## Introduction

Charged particle therapy, including proton therapy and heavy ion therapy, has gained increasing attention in oncology due to its superior dose distribution and radio-biological effectiveness (RBE) compared to conventional photon radiotherapy.^1^, ^2^ The physical properties of charged particles, particularly the Bragg peak phenomenon, allow for precise dose deposition within the tumor while minimizing damage to surrounding healthy tissues.^3^, ^4^ Particle therapy, particularly with heavier ions such as carbon, helium, etc, also has higher radio-RBE. These characteristics are particularly advantageous for treating deep-seated and/or radio-resistant tumors, as well as selected group of patients, such as pediatric patients, where reducing long-term radiation toxicity is critical.^5^ Despite these benefits, particle therapy poses unique challenges in treatment planning, requiring highly accurate dose calculations, robust optimization techniques, and consideration of modeling biological effects such as linear energy transfer (LET) and relative RBE.^6^, ^7^, ^8^

Treatment planning in particle therapy is a complex and computationally intensive process, involving tumor delineation, beam arrangement, dose calculation, and plan optimization to achieve the best possible therapeutic outcome.^9^ Unlike conventional photon therapy, where dose deposition follows a more predictable pattern, particle therapy is highly sensitive to anatomical variations, beam uncertainties, and biological factors that affect treatment efficacy.^10^ As a result, developing efficient, robust, and adaptive treatment planning strategies is essential to maximize the clinical benefits of particle therapy while ensuring treatment accuracy and safety.^11^

Artificial intelligence (AI) has emerged as a promising tool to streamline and enhance treatment planning in radiotherapy, including particle therapy.^12^, ^13^ Recent advances in machine learning (ML) and deep learning (DL) have enabled the automation of key planning steps, such as target segmentation, dose prediction, and plan adaptation, potentially reducing planning time and inter-clinician variability.^14^, ^15^, ^16^ AI-based algorithms are also being explored for improving robust optimization, biological modeling, and adaptive treatment strategies in response to anatomical changes.^17^ Despite these advancements, challenges remain regarding data availability, model interpretability, and clinical validation, which must be addressed before AI-driven approaches can be fully integrated into routine clinical practice.^18^, ^19^, ^20^

This review aims to provide a comprehensive overview of AI applications in particle therapy treatment planning, summarizing recent advancements, ongoing challenges, and future research directions. Specifically, we discuss AI-driven dose calculation, automated treatment planning, adaptation techniques, and quality assurance approaches. Moreover, we highlight the limitations and clinical considerations associated with implementing AI in particle therapy workflows. By examining the current state of AI in this field, this review seeks to identify key areas for future development and facilitate the translation of AI-based methodologies into clinical practice.

### Fundamentals of artificial intelligence in treatment planning

The integration of AI into treatment planning for particle therapy is increasingly being explored to enhance automation, efficiency, and accuracy. AI-driven methodologies are largely based on ML and DL, offering solutions for complex tasks such as target segmentation, dose distribution prediction, and plan optimization.^21^, ^22^ By using large datasets and computational models, AI has the potential to improve treatment planning workflows and personalize therapy for individual patients.^23^ However, challenges related to data availability, model interpretability, and clinical validation must be addressed for widespread adoption.^24^

ML approaches in treatment planning include both conventional algorithms, such as support vector machines and decision trees,^25^ and more advanced DL architectures like convolutional neural networks (CNNs) and transformer-based models. CNNs have demonstrated success in automating tumor and organ-at-risk segmentation, reducing inter-observer variability and planning time.^26^, ^27^ DL models have also been used to predict dose distributions based on historical treatment data, providing rapid estimations without requiring full physics-based calculations.^28^ Moreover, recurrent neural networks^29^ and long short-term memory (LSTM) networks^30^ have been applied to track anatomical changes over time, allowing for adaptive planning in response to patient-specific variations. Despite these advancements, DL models require large, high-quality datasets for training, and their black-box nature poses challenges for clinical validation and trustworthiness.

Optimization is a crucial component of treatment planning, where AI-driven methods aim to improve dose distribution efficiency and computational speed. Traditional optimization techniques, such as gradient-based methods and stochastic approaches, are being supplemented with AI-based strategies.^31^, ^32^ Reinforcement learning (RL) has emerged as a promising technique, where an AI agent learns optimal treatment parameters through iterative interactions with a simulated planning environment.^33^ Evolutionary algorithms, including genetic algorithms and particle swarm optimization, have also been explored for optimizing beam arrangements and fluence maps, particularly in the presence of anatomical or setup uncertainties.^34^ Another emerging approach involves hybrid AI-physics models, which integrate DL techniques with physics-based dose calculations to enhance both accuracy and computational efficiency.^35^ While these AI-driven optimization methods have demonstrated potential in reducing planning time and improving plan quality, ensuring their robustness and clinical acceptability remains a challenge.

AI-based treatment planning can be broadly categorized into data-driven models^36^ and physics-based models.^37^ Data-driven models rely on large datasets to predict treatment parameters, often using DL techniques to generate dose distributions or optimize beam configurations. These models can significantly reduce the computational burden associated with conventional planning; however, their reliance on historical data raises concerns about generalizability across diverse patient populations.^38^ In contrast, physics-based models use established dose calculation algorithms, such as Monte Carlo simulations or pencil beam algorithms, to predict dose deposition with high accuracy. While physics-based models offer greater interpretability, they are computationally expensive and less adaptable for real-time applications.^39^ A hybrid approach combining AI-based learning with physics-based principles is emerging as a promising solution, aiming to balance computational efficiency with the accuracy and reliability required for clinical implementation.

### Artificial intelligence applications in particle therapy treatment planning

The application of AI in particle therapy treatment planning has gained increasing attention due to its potential to improve efficiency, accuracy, and adaptability. AI-driven approaches aim to enhance various aspects of treatment planning, from automated plan generation and dose optimization to real-time plan adaptation. These applications employ ML and DL techniques to streamline traditionally complex and labor-intensive processes. However, integrating AI into clinical workflows requires overcoming challenges related to data availability, model interpretability, and regulatory approval.

#### Automated plan generation

One of the key areas where AI is making an impact is automated treatment plan generation, which involves optimizing key parameters such as beam angles, dose distribution, and fractionation schedules.^40^ AI-based parameter selection methods use historical treatment data and ML algorithms to identify optimal plan settings, reducing the need for extensive manual adjustments.^41^ Supervised learning techniques have been used to develop predictive models that assist in selecting parameters based on patient-specific anatomical and clinical features.^42^ This automation not only improves efficiency but also ensures consistency in plan quality across different treatment centers.

**Figure 1:**
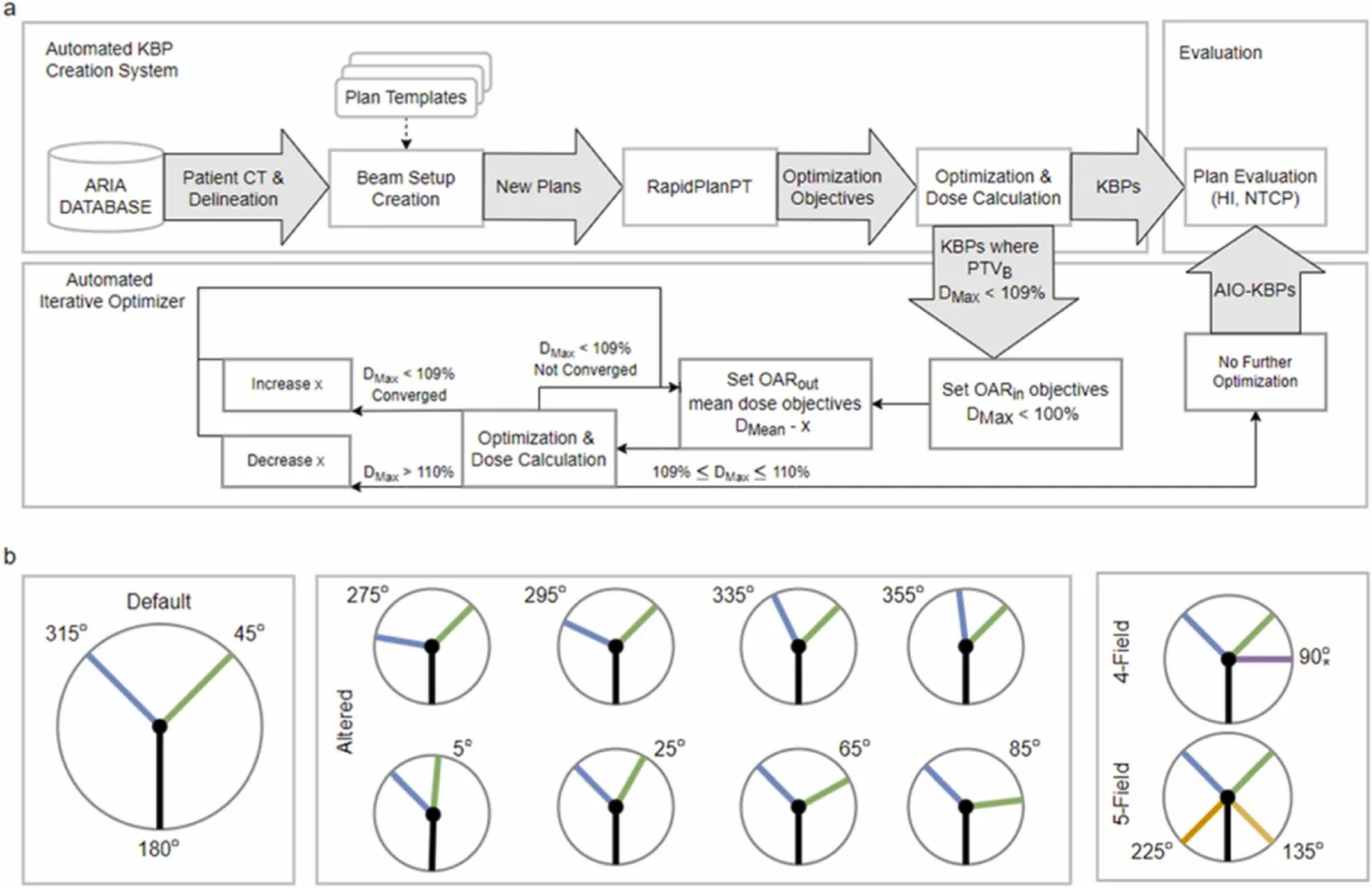
(a) Workflow for KBP creation and AIO processing. (b) Beam angle arrangement illustration. Angles not separately indicated in the middle or right-pane diagrams are the same as in the default arrangement. KBP, knowledge-based plan; AIO, automatic iterative optimizer; HI, homogeneity index; NTCP, normal tissue complication probability; OAR, organ at risk.^43^

Another critical aspect of automated plan generation is beam arrangement and dose calculation, where AI models help optimize beam angles and energy levels to maximize tumor coverage while minimizing dose to surrounding healthy tissues.^43^ Deep RL has been explored for dynamic beam selection, enabling AI agents to iteratively refine treatment plans by simulating different beam arrangements and assessing their dosimetric impact.^44^ In addition, neural networks have been employed to accelerate dose calculation processes, replacing traditional computationally expensive methods with fast, data-driven approximations.^45^ These advancements contribute to a more efficient planning workflow, reducing the time required to generate clinically acceptable plans. Figure 1(a) illustrates the workflow for creating a knowledge-based plan using an automatic iterative optimizer with various beam angles in proton therapy. Figure 1(b) shows the beam angle arrangement in the plan,^43^ demonstrating how AI can enhance beam geometry in a proton treatment plan.

#### Dose prediction and optimization

AI has also been applied to dose distribution modeling and optimization, offering new possibilities for improving treatment accuracy and efficiency. Conventional dose calculation methods rely on physics-based models that require significant computational resources.^46^ In contrast, AI-driven dose prediction models use DL techniques to rapidly estimate 3-dimensional dose distributions based on https://www.mdpi.com/cancers/cancers-14–02849/article_deploy/html/images/cancers-14–02849-g001.png prior patient data.^47^ CNNs have demonstrated the ability to accurately predict dose distributions within seconds, providing an efficient alternative to Monte Carlo-based calculations.^48^

Beyond standard dose distribution modeling, AI is being explored to account for biological factors such as LET and RBE, which are particularly important in heavy ion therapy.^49^ LET and RBE influence the biological impact of radiation on tissues, making their accurate estimation essential for optimizing treatment plans. AI-based approaches have been proposed to predict LET and RBE distributions using patient-specific imaging and treatment parameters, potentially improving the precision of biologically optimized treatment plans.^50^, ^51^ However, challenges remain in validating these models across different particle therapy systems and clinical scenarios.

#### Treatment plan adaptation

One of the key advantages of AI in particle therapy is its ability to facilitate treatment plan adaptation, addressing anatomical and physiological changes that occur over the course of treatment. AI models can predict anatomical variations by analyzing patient imaging data collected before and during treatment.^52^ ML algorithms have been developed to anticipate tumor shrinkage, organ motion, and weight loss, allowing for proactive plan modifications.^53^ Such predictive capabilities can help clinicians adjust treatment parameters in response to patient-specific changes, improving plan robustness and treatment efficacy.

In addition to predictive modeling, AI plays a role in real-time adaptive re-planning, where treatment plans are dynamically updated based on intrafractional or interfractional changes.^54^ DL models have been trained to detect and quantify anatomical shifts in daily imaging scans, enabling automated plan adjustments without requiring manual intervention.^55^ AI-driven adaptive planning is particularly relevant for proton and heavy ion therapy, where small variations in patient positioning can significantly affect dose distribution.^56^ Although promising, real-time adaptive re-planning remains an area of active research, with ongoing efforts to refine model accuracy, computational efficiency, and clinical implementation strategies.

Table 1 provides a comprehensive summary of the various AI applications in particle therapy treatment planning. Overall, AI-driven applications in particle therapy treatment planning offer significant potential to improve plan quality, efficiency, and adaptability. While notable progress has been made, further research is needed to validate AI models across diverse clinical settings and integrate them into routine practice. Addressing challenges such as data standardization, model interpretability, and regulatory approval will be crucial for the successful translation of AI-based treatment planning into clinical workflows.

**Table 1:** Summary of AI applications in particle therapy treatment planning.

Application Area	Description	References
Automated Plan Generation	− Optimizes beam angles, dose distribution, and fractionation schedules using historical data and ML algorithms.	40, 41, 42
	− Uses deep RL for dynamic beam selection and neural networks for fast dose calculation.	43, 44, 45
Dose Prediction and Optimization	− Employs DL techniques for rapid 3D dose distribution estimation, offering an efficient alternative to traditional methods.	46, 47, 48
	− Accounts for biological factors like LET and RBE, improving precision in heavy ion therapy.	49, 50, 51
Treatment Plan Adaptation	− Predicts anatomical variations using patient imaging data, allowing for proactive plan modifications.	52, 53
	− Facilitates real-time adaptive re-planning based on intrafractional or interfractional changes, particularly relevant for proton and heavy ion therapy.	54, 55, 56

### Clinical implementation and validation

**Figure 2:**
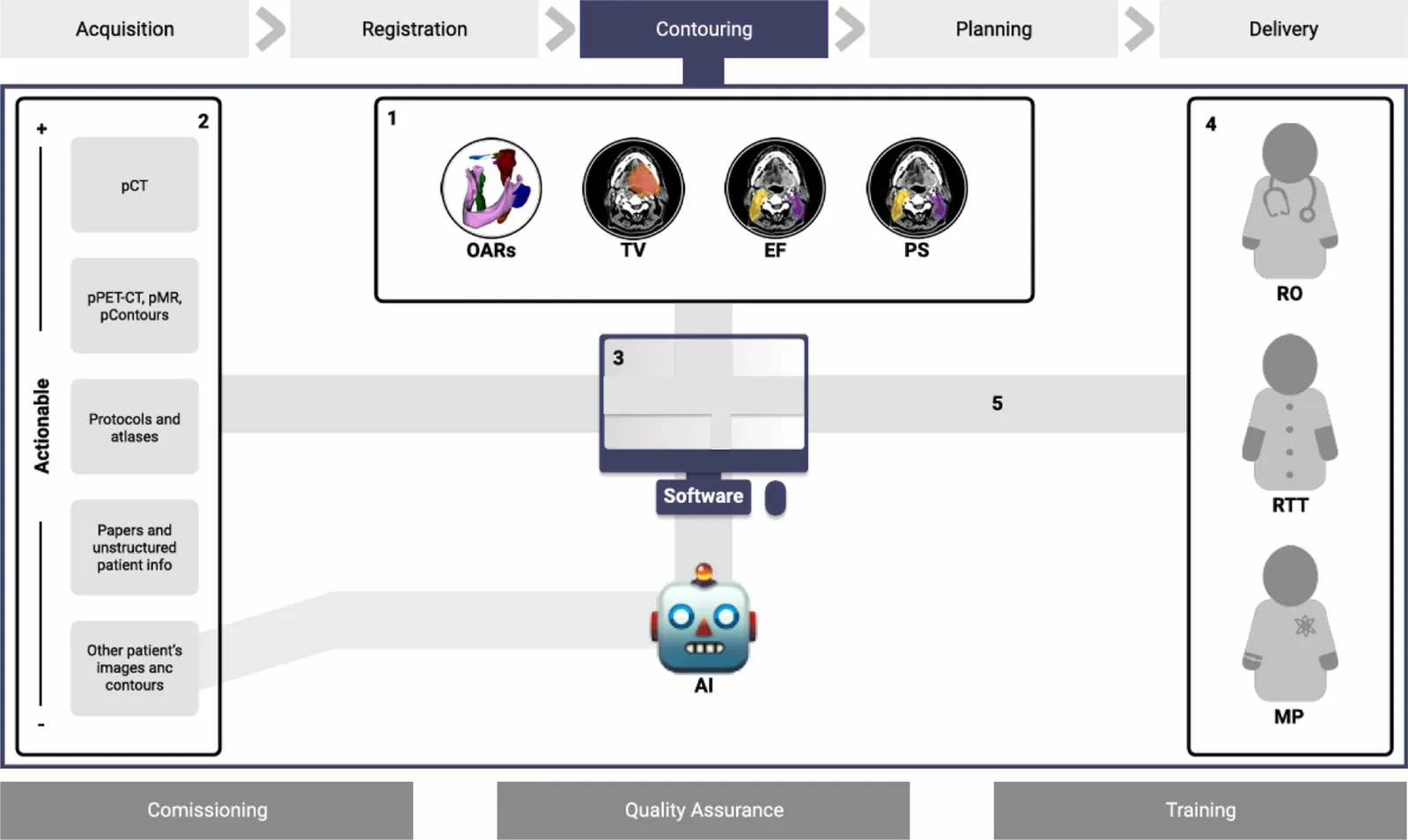
An activity theory analysis of the interview and observational data identified 5 key aspects central to contouring: (1) the activity's objective, (2) data sources, (3) software, (4) RT practitioners, and (5) the hierarchical structure of the activity and performance factors.^58^

The integration of AI into particle therapy treatment planning requires thorough clinical validation and regulatory approval before widespread adoption. While AI-driven methods have demonstrated promising results in automating treatment planning, dose optimization, and plan adaptation, their clinical applicability depends on their performance compared to conventional approaches, quality assurance (QA) measures, and compliance with regulatory standards.^57^, ^58^ Addressing these factors is essential to ensure that AI-based models enhance clinical workflows without compromising patient safety or treatment efficacy. For example, Figure 2 illustrates the clinical auto-contouring workflow in AI-assisted treatment planning at a proton center. Five key aspects play a central role in contouring: (1) the activity's objective, (2) data sources, (3) software, (4) RT practitioners, and (5) the hierarchical structure of the activity and performance factors.

#### Performance comparison: Artificial intelligence versus conventional planning

A critical step in validating AI-driven treatment planning is comparing its performance against conventional planning methods. Traditional treatment planning relies on manual adjustments and physics-based dose calculations, which, while well-established, are time-consuming and subject to inter-operator variability.^59^ AI-driven planning, on the other hand, offers the potential for increased efficiency by automating key steps such as beam arrangement, dose prediction, and plan optimization.

Several studies have shown that AI-generated treatment plans can achieve comparable or superior dosimetric outcomes while significantly reducing planning time.^60^, ^61^, ^62^ DL-based dose prediction models have demonstrated accuracy comparable to Monte Carlo simulations but with a fraction of the computational cost. RL-based beam arrangement strategies have been shown to optimize treatment plans with minimal human intervention.^63^ However, while AI-based approaches can enhance efficiency and consistency, their ability to handle complex, patient-specific variations in anatomy and biological response remains an area of ongoing research. Further clinical trials and large-scale retrospective analyses are needed to establish AI’s superiority or equivalence to conventional methods.^64^

#### AI-based quality assurance and validation techniques

Ensuring the reliability and safety of AI-generated treatment plans is a key requirement for clinical implementation. AI-based QA techniques have been developed to assess the accuracy of treatment plans by incorporating additional validation steps, such as uncertainty quantification, anomaly detection, and independent dose verification.^65^, ^66^ ML models trained on historical treatment data can identify deviations in planned dose distributions, flagging potentially suboptimal or unsafe plans before clinical approval.

Another approach involves explainable AI (XAI) techniques, which aim to improve model interpretability by providing insights into how AI algorithms make decisions.^67^ This is particularly important in a clinical setting, where black-box AI models may not be fully trusted by clinicians. AI-based QA tools can also be integrated into automated treatment verification systems, continuously monitoring the accuracy of delivered doses by comparing predicted and measured treatment parameters.^68^

Despite these advancements, one of the major challenges in AI validation is ensuring robustness across different clinical settings. AI models trained on data from a specific institution or treatment machine may not generalize well to other centers due to variations in imaging protocols, machine calibration, and patient demographics. To address this, multi-institutional validation studies and standardized benchmarking datasets are needed to assess model performance across diverse clinical environments.^69^, ^70^

#### Regulatory and clinical acceptance challenges

The adoption of AI in clinical practice is subject to regulatory approval and adherence to safety guidelines established by organizations such as the U.S. Food and Drug Administration, the European Medicines Agency, and the International Atomic Energy Agency.^71^, ^72^ Unlike traditional treatment planning algorithms, which rely on deterministic physics-based calculations, AI-driven methods often involve probabilistic models that require extensive validation before regulatory clearance. The lack of standardized evaluation criteria for AI in radiotherapy presents an additional hurdle, as different institutions may employ varying methodologies for testing AI algorithms.

Another key challenge is clinical acceptance and trust in AI-driven treatment planning. Clinicians and medical physicists must have confidence in AI-generated plans, particularly in scenarios where the model’s decision-making process is not fully transparent.^73^, ^74^ Training programs and AI literacy initiatives are essential to ensure that healthcare professionals understand the strengths and limitations of AI-based planning.^75^ Moreover, human-in-the-loop approaches, where AI-generated plans are reviewed and adjusted by clinical experts, may help facilitate acceptance and integration into routine workflows.^76^

Despite these challenges, the potential benefits of AI in treatment planning—including increased efficiency, reduced variability, and enhanced adaptability—justify ongoing research and development efforts. Establishing robust validation frameworks, improving model transparency, and addressing regulatory concerns will be crucial for the successful clinical implementation of AI in particle therapy treatment planning.

### Challenges and future directions

Despite the significant progress in AI-driven treatment planning for particle therapy, several challenges must be addressed before these approaches can be widely adopted in clinical practice. Issues related to data availability, model generalization, ethical considerations, and AI explainability remain major obstacles.^77^ Additionally, future research should focus on hybrid AI-physics models, RL, and federated learning to enhance AI’s robustness and applicability in treatment planning.^78^, ^79^

#### Data availability and model generalization issues

AI models in treatment planning rely on large, high-quality datasets to learn complex relationships between patient anatomy, dose distribution, and treatment outcomes. However, access to diverse, well-annotated datasets is often limited due to data privacy regulations, institutional restrictions, and variations in treatment protocols across different centers.^80^, ^81^ The lack of standardized datasets makes it difficult to develop AI models that generalize well across diverse patient populations and clinical settings.^82^

Moreover, AI models trained on data from a single institution may not perform optimally when applied to new patient cohorts due to differences in imaging modalities, machine calibration, and clinical workflows. This lack of generalizability raises concerns about the reliability of AI-generated treatment plans in real-world clinical practice. Addressing this issue requires multi-institutional collaborations, data-sharing frameworks, and standardized benchmarking datasets to improve model robustness and ensure broader applicability.^83^, ^84^

#### Ethical concerns and explainability in artificial intelligence decisions

The use of AI in treatment planning introduces ethical concerns related to bias, transparency, and accountability. AI models trained on limited or non-representative datasets may inadvertently introduce biases, leading to suboptimal treatment recommendations for certain patient groups.^85^, ^86^ Ensuring fairness and equity in AI-driven treatment planning is critical, particularly in cases where model predictions influence clinical decisions.

Another major concern is the lack of explainability in AI-based treatment planning. Many DL models operate as "black boxes," making it difficult for clinicians to understand how treatment recommendations are generated. This lack of transparency can lead to skepticism among medical professionals, potentially hindering clinical adoption. XAI techniques, such as saliency maps,^87^ feature attribution methods,^88^ and interpretable ML models,^89^ are being explored to improve model transparency and build trust in AI-driven decision-making.

In addition, legal and regulatory frameworks for AI in medicine are still evolving. Questions regarding liability and accountability in AI-assisted treatment planning need to be addressed, particularly in cases where AI-generated plans deviate from standard clinical practices.^90^ Establishing clear guidelines for AI validation, clinical oversight, and regulatory approval will be essential for ethical and responsible implementation.^71^, ^91^

#### Future research directions: Hybrid artificial intelligence -physics models, reinforcement learning , federated learning, and quantum machine learning

To overcome existing limitations and improve the clinical applicability of AI in particle therapy treatment planning, future research should focus on hybrid AI-physics models, RL, federated learning approaches, and quantum ML (QML).

**Figure 3:**
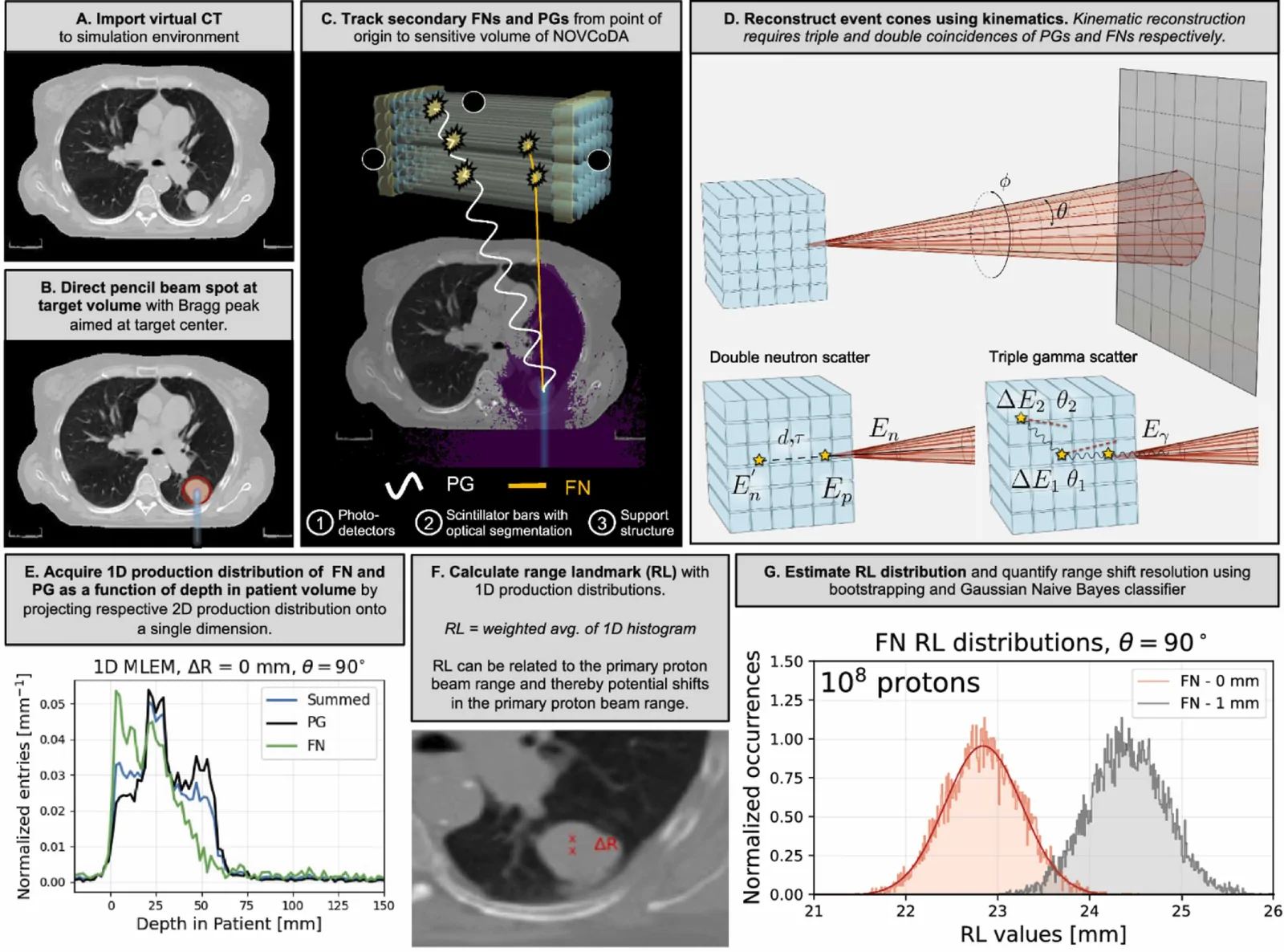
The NOVCoDA design and analysis workflow: (A) A virtual 3D patient model with non-small cell lung cancer is imported into the Monte Carlo simulation environment. (B) A mono-energetic proton pencil beam targets the volume. (C) Secondary FNs and PGs are tracked through the patient geometry and NOVCoDA's sensitive volume. (D) Triple and double coincidences enable kinematic reconstruction of event cones, which are back-projected and followed by image reconstruction into 2D FN and PG distributions. (E) These 2D distributions are projected into 1D histograms along the proton beam direction. (F) The 1D distributions calculate a range landmark (RL) parameter related to the proton beam range and potential range shifts. (G) A bootstrapping procedure with a Gaussian Naive Bayes classifier estimates range landmark distributions and quantifies NOVCoDA's range-shift-resolving capabilities.^92^

Hybrid AI-Physics Models: Combining AI-based learning with physics-based dose calculation models can enhance the accuracy and reliability of treatment planning.^92^ While pure DL models can approximate dose distributions with high speed, they may lack the physical consistency required for clinical acceptance. Hybrid approaches, which integrate AI-driven predictions with Monte Carlo simulations or analytical dose models, can balance computational efficiency with physical accuracy, improving the clinical feasibility of AI-assisted planning.^93^, ^94^ Figure 3 shows a hybrid system (NOVCoDA) combining measurements of prompt-gamma rays (PG) and fast neutrons (FN) for range probing in particle treatment verification.

RL: RL has shown promise in optimizing treatment planning by allowing AI agents to iteratively learn optimal treatment strategies through trial and error. In particle therapy, RL-based models could be used to dynamically adjust beam arrangements, dose fractionation schedules, and treatment adaptations based on real-time patient responses.^95^, ^96^ However, further research is needed to refine RL algorithms for clinical use and ensure their reliability in highly variable treatment scenarios.

Federated Learning: Data privacy concerns have been a major barrier to developing robust AI models in radiotherapy.^97^ Federated learning offers a solution by enabling multiple institutions to collaboratively train AI models without sharing raw patient data. Instead, AI models are trained locally on institutional data, and only model updates (rather than patient information) are exchanged.^98^ This approach can improve model generalization and performance across diverse clinical settings while preserving patient privacy. However, federated learning requires standardization of data formats and computational infrastructure to ensure interoperability across institutions.

QML: The application of quantum computing in ML (QML) represents an emerging frontier in AI-driven treatment planning.^99^ Quantum algorithms have the potential to significantly accelerate complex optimization problems, such as beam angle selection and dose distribution calculations, which are computationally demanding in conventional approaches.^100^ Quantum-enhanced RL could be explored for optimizing adaptive treatment planning, where a vast number of possible treatment adjustments must be evaluated in real-time.^101^ In addition, hybrid quantum-classical AI models could improve the efficiency of Monte Carlo simulations by leveraging quantum computing’s ability to process high-dimensional data and simulate particle interactions more efficiently.^102^, ^103^ While QML remains in its early stages, ongoing advancements in quantum hardware and algorithm development may enable its future integration into radiotherapy planning, providing a computational advantage in solving highly complex, data-intensive problems.^104^

Table 2 summarizes key challenges and future directions in AI-driven particle therapy planning, including data limitations, ethical concerns, and regulatory needs. As AI continues to evolve, addressing these challenges and exploring new research directions will be critical to advancing its role in particle therapy treatment planning. Future efforts should focus on developing clinically validated AI models, enhancing transparency and trust, and integrating AI seamlessly into clinical workflows to maximize its potential benefits for patient care.

**Table 2:** Challenges and future directions in AI-driven particle therapy treatment planning.

Category	Challenges and Future Directions	References
Data Availability & Model Generalization	− Limited access to diverse, well-annotated datasets due to privacy regulations and institutional restrictions. − AI models trained on single-institution data may lack generalizability across different clinical settings. − Need for multi-institutional collaborations and standardized benchmarking datasets.	77, 80, 81, 82, 83, 84
Ethical Concerns & Explainability	− AI models may introduce biases if trained on non-representative datasets, affecting treatment recommendations. − Lack of transparency in AI decisions ("black box" problem) reduces clinical trust. − Need for XAI techniques like saliency maps and interpretable ML models. − Legal and regulatory challenges in AI validation and accountability.	85, 86, 87, 88, 89, 90, 91
Hybrid AI-Physics Models	− Combining AI with physics-based dose calculation can improve accuracy and reliability. − Hybrid models integrating Monte Carlo simulations can ensure physical consistency while maintaining computational efficiency.	92, 93, 94
Reinforcement Learning (RL)	− RL can optimize beam arrangements, dose fractionation schedules, and adaptive treatment strategies. − Requires further research to refine algorithms and ensure reliability in dynamic treatment scenarios.	95, 96
Federated Learning	− Enables AI model training across multiple institutions without sharing raw patient data, improving generalization. − Requires standardization of data formats and computational infrastructure for interoperability.	97, 98
Quantum Machine Learning (QML)	− Quantum computing can accelerate optimization tasks like beam angle selection and dose distribution calculations. − Potential for quantum-enhanced RL in adaptive treatment planning. − Hybrid quantum-classical AI models could improve Monte Carlo simulations for radiotherapy.	99, 100, 101, 102, 103, 104

## Conclusion

The integration of AI into particle therapy treatment planning has the potential to enhance efficiency, accuracy, and adaptability in clinical workflows. AI-driven approaches, including DL, RL, and hybrid AI-physics models, have demonstrated promising results in automating plan generation, dose optimization, and adaptive treatment strategies. These advancements aim to improve plan quality while reducing the time and effort required for manual adjustments, thereby supporting clinicians in delivering more precise and personalized treatments.

Despite these benefits, several challenges must be addressed before AI-based treatment planning can be widely implemented in clinical practice. Issues related to data availability, model generalization, and explainability remain significant obstacles. Ensuring the transparency and interpretability of AI models is critical for gaining clinical trust and regulatory approval. Moreover, the ethical and legal implications of AI-driven decision-making in radiotherapy require further consideration, particularly in terms of accountability and patient safety.

Future research should focus on overcoming these limitations by exploring innovative AI methodologies, such as federated learning for privacy-preserving model training and quantum ML for enhanced computational efficiency. Establishing standardized validation frameworks and conducting multi-institutional studies will be essential for demonstrating the reliability and clinical utility of AI-driven treatment planning systems.

As AI continues to evolve, its role in particle therapy treatment planning will likely expand, offering new opportunities to optimize radiation delivery and improve patient outcomes. However, successful integration into clinical practice will require a multidisciplinary effort involving researchers, clinicians, regulatory bodies, and technology developers. By addressing current challenges and leveraging emerging AI capabilities, the field can move toward a future where AI-driven treatment planning becomes a standard component of precision radiation oncology.

## CRediT authorship contribution statement

James Chow: Methodology, Investigation, Formal analysis, Visualization, Writing – original draft. Huan Giap: Conceptualization, Project Administration, Funding acquistion, Writing - reveiw and editing.

## Declaration of Competing Interest

The authors declare that they have no known competing financial interests or personal relationships that could have appeared to influence the work reported in this paper.
